# Development of the football tactical skills scale and comparison of scores across player and training characteristics

**DOI:** 10.1186/s13102-026-01934-z

**Published:** 2026-08-01

**Authors:** Oğuzhan Eroğlu, Alper Karadağ

**Affiliations:** 1 Sports Management Department, Muş Alparslan University Varto Vocational School, Muş, 49000 Turkey; 2 Faculty of Sports Sciences, Muş Alparslan University, Muş, Turkey

**Keywords:** Football, Tactical skills, Scale development, Self-report, Decision-making

## Abstract

**Background:**

Tactical performance in football emerges from the interaction of perceptual-cognitive processes, decision-making, positional responsibilities, psychological regulation, and coordinated action under changing game constraints. Although tactical behaviour can be examined through observational and game-based procedures, there remains a need for a standardized, practically administered self-report instrument that captures how players evaluate their own tactical actions across different developmental and competitive contexts. This study aimed to develop and initially validate the Football Tactical Skills Scale (FTSS) and to examine whether FTSS scores differed according to selected player, training, and psychosocial characteristics.

**Methods:**

The study used a cross-sectional survey and scale-development design. The scale-development process included a pilot study (*n* = 50), exploratory factor analysis (*n* = 320), and confirmatory factor analysis (*n* = 320). Group comparisons were conducted in a sample of 700 football players aged 14 years and older (x̄_age_=18.22 ± 4.60). Content validity, construct validity, internal consistency, lower-upper group discrimination, and convergent and discriminant validity were examined. Descriptive statistics, one-way analysis of variance, independent-samples t-tests, Tamhane’s T2 post-hoc tests, eta-squared, and Cohen’s d were used in the comparative analyses.

**Results:**

The 17-item FTSS comprised four dimensions: game reading and decision-making, psychological skills and game discipline, transitions in game and field utilization, and strategic thinking and risk management. The factor structure showed acceptable-to-excellent fit and satisfactory reliability and validity indicators. Higher self-reported FTSS scores were observed among older players, players competing at higher league levels, players reporting more weekly training sessions, mental training, visual support training, and better communication with coaches. Scores also differed by playing position; midfielders obtained the highest total scores, whereas wingers obtained the highest scores in strategic thinking and risk management (*p*<0.001).

**Conclusions:**

The findings provide initial support for the FTSS as a multidimensional self-report questionnaire for assessing players’ perceived tactical skills in football. The scale was sensitive to systematic differences across age, position, competitive level, training exposure, and selected psychosocial characteristics. Because the study was cross-sectional and did not include an external criterion of observed tactical performance, FTSS scores should not be interpreted as direct measures of match behaviour or as evidence of causal effects. Future research should examine test-retest reliability, measurement invariance, cross-cultural validity, and criterion validity against tools such as FUT-SAT, GPAI, coach ratings, notational analysis, and representative small-sided games.

**Supplementary Information:**

The online version contains supplementary material available at https://doi.org/10.1186/s13102-026-01934-z.

## Introduction

Football requires players to coordinate technical execution, physical effort, and tactical action in a dynamic environment characterized by time pressure, spatial constraints, uncertainty, and continuous interaction with teammates and opponents. Effective performance therefore depends not only on executing a skill, but also on perceiving relevant information, anticipating how play may develop, selecting an appropriate response, and adapting that response as the situation changes [1–4]. Tactical behaviour can be understood as the functional organization of individual and collective actions in relation to the objectives and constraints of the game. It is expressed through positioning, support, cover, the creation and use of space, transitions between attack and defence, and coordinated decision-making. These behaviours are closely related to perceptual-cognitive skills and tactical intelligence because players must identify relevant cues, recognize patterns, anticipate opponents’ actions, and select solutions that are timely and consistent with the team’s tactical principles [5–7].

Tactics and tactical skills are related but distinct concepts. Tactics refer to the principles and strategies used by individuals and teams to achieve game objectives, whereas tactical skills refer to a player’s capacity to interpret the game and apply appropriate actions at the right time [8, 9]. Tactical skills encompass both declarative knowledge of what to do and procedural understanding of how and when to act. However, the Football Tactical Skills Scale (FTSS) primarily focuses on players’ self-reported application of these capacities in game-related situations. Tactical functioning involves the integration of perception, anticipation, decision-making, positioning, risk evaluation, emotional regulation, and coordinated execution under representative game conditions [1, 2, 5]. In the present study, perceived tactical skills are operationalized as a multidimensional self-report construct comprising four interrelated domains: game reading and decision-making; psychological skills and game discipline, transitions in game and field utilization; and strategic thinking and risk management. Within this operationalization, psychological skills and game discipline refer to game-specific self-regulation that supports the enactment of tactical intentions under pressure; they are not intended to represent or replace general psychological constructs.

An important distinction must be made between observed tactical performance and self-reported tactical skills. Tactical behaviour can be assessed directly through match observation, notational and positional analyses, video-based decision-making tasks, small-sided games, and game-performance instruments. The System of Tactical Assessment in Soccer (FUT-SAT), evaluates tactical actions performed in game situations according to core tactical principles [10], whereas the Game Performance Assessment Instrument (GPAI) examines components such as decision-making, support, adjustment, and skill execution during game play [11]. Representative learning design further emphasizes that assessment and practice tasks should preserve the informational constraints and action possibilities of competition [12]. These approaches provide valuable evidence about what players actually do; however, they may require trained observers, video recording, coding procedures, appropriate facilities, or repeated game-based testing, which can limit their feasibility in large and heterogeneous samples [4, 13, 14].

Self-report instruments address a different but complementary question: how players understand and evaluate their own tactical functioning. The Tactical Skills Inventory for Sports and subsequent studies have demonstrated the usefulness of self-assessment for examining perceived positioning, decision-making, knowledge of ball actions, and tactical functioning across performance levels [1, 15, 16]. Such instruments can be administered efficiently to large groups, can capture experiences that may not be visible in a single observation, and may reveal discrepancies between players’ self-perceptions and the evaluations of coaches or analysts. Nevertheless, self-reported scores may also be influenced by confidence, social desirability, response style, or limited self-awareness and therefore cannot be assumed to correspond directly to objective tactical performance [17]. Accordingly, the FTSS was designed to assess players’ self-reported or perceived tactical skills in game-related situations. It is intended to complement rather than replace FUT-SAT, GPAI, expert observation, notational analysis, or representative game tasks. External criterion validity must therefore be established before FTSS scores can be interpreted as indicators of observed match behaviour.

Perceived tactical skills may also vary across developmental, competitive, positional, and training contexts. With increasing age and experience, players are exposed to more complex tactical content, a wider range of game situations, and greater demands for anticipation and decision-making [16, 18, 19]. Competitive level may reflect both accumulated learning opportunities and selection processes, whereas playing positions impose different spatial, physical, and decision-making demands. Tactical action is therefore not independent of physical capacity or movement behaviour. The ability to occupy space, support play, press, recover, and manage transitions is constrained by running demands, available space, role-specific responsibilities, and the speed at which game states change [20–22]. Mental preparation, visual or video-based support, and coach-player communication may also be associated with perceived tactical skills through their relationships with attentional control, anticipation, feedback processing, shared understanding, and confidence. However, such relationships should be interpreted as associations unless longitudinal or experimental evidence demonstrates causal effects.

Despite the increasing number of approaches used to assess tactical knowledge and performance in football, the literature remains conceptually and methodologically fragmented. A scoping review identified 29 tactical assessment tests across 24 studies and reported substantial terminological variation, with 12 different terms used to describe the tactical domain; only a limited number of instruments met most of the proposed quality criteria [23]. Similarly, a systematic review of scenario-based declarative tactical knowledge tools found that many instruments did not satisfy several methodological quality criteria and that few provided specific scores based on tactical principles or player subroles [24]. Broader reviews of youth football skill assessment have also shown considerable variation in assessment procedures, frequent use of tasks that do not adequately represent match play, and a predominant emphasis on offensive skill outcomes [25]. Collectively, these findings indicate that the principal limitation is not the complete absence of tactical assessment tools, but the heterogeneity of their terminology, task design, target constructs, populations, and psychometric support.

Existing instruments also address different aspects of tactical functioning. Observational systems such as FUT-SAT and the Tactical Assessment Instrument in Football provide ecologically relevant information about players’ actions in game situations but require systematic observation, trained assessors, and coding procedures [10, 26]. Video- and scenario-based tools primarily assess decision-making or declarative tactical knowledge, whereas available self-report measures differ in scope and target population. The Tactical Skills Inventory for Sports was developed for both youth football and field hockey and assesses general declarative and procedural tactical knowledge across team sports [15]. The Perceived Game-Specific Soccer Competence Scale is football-specific but was developed for players aged 12–15 years and focuses mainly on perceived offensive, one-on-one, and defensive competence [27]. Moreover, self-reported tactical knowledge may not correspond directly to tactical behaviour observed in representative game situations [17]. Therefore, there remains a specific need for a brief football-specific self-report instrument that can be administered across a broader developmental and competitive range and that integrates game reading and decision-making, psychological skills and game discipline, transitions in game and field utilization, and strategic thinking and risk management within a single multidimensional structure. Such an instrument should be regarded as complementary to, rather than a replacement for, observational and representative performance assessments.

A large and heterogeneous validation design is valuable for two reasons. First, exploratory and confirmatory analyses conducted in independent samples reduce the likelihood that the identified factor structure is sample-specific and provide a stronger initial evaluation of its reproducibility. Second, including players from different age groups, playing positions, competitive levels, and training contexts permits a preliminary examination of whether FTSS scores vary across theoretically relevant player and training characteristics. These comparisons do not establish measurement invariance, criterion validity, or causality. However, they can strengthen the initial evaluation of the scale and guide subsequent validation research.

Accordingly, the aims of this study were: (1) to develop the Football Tactical Skills Scale and examine its content validity, factor structure, internal consistency, convergent validity, discriminant validity, and lower–upper group discrimination; and (2) to compare FTSS scores across age groups, playing positions, competitive levels, weekly training frequency, reported participation in mental training, reported exposure to visual support training, and perceived coach communication quality. Because the comparative component used a cross-sectional design, the examined characteristics were treated as variables associated with FTSS scores rather than as causal determinants of perceived tactical skills.

## Method

### Research model

The study combined a scale-development design with a cross-sectional survey design. Independent samples were used for the exploratory and confirmatory stages of scale development, and a separate 700-player dataset was used to examine score distributions, internal consistency, and group differences. Because the demographic and training variables and FTSS scores were measured at one time point, the analyses identify associations and group differences but do not establish temporal or causal effects.

### Population and sample

The study’s sample group consisted of football players aged 14 and older who were actively playing in football leagues in Turkiye during the 2024–2025 season. In this study, data were collected from different study groups at different time points. The scale development working group comprised 50 football players for the pilot study, 320 for exploratory factor analysis, and 320 for confirmatory factor analysis. The sample used to examine FTSS score differences across player, training, and communication characteristics consisted of 700 football players. The demographic characteristics of the participants are presented in Table 1.

**Table 1:** Statistical distributions of participants’ demographic characteristics

Variables	Groups	*n*	%
Age	Age 14–15	233	33.3
	Age 16–17	187	26.7
	Age 18–19	123	17.6
	Ages 20 and older	157	22.4
Position	Goalkeeper	78	11.1
	Right Back / Left Back	94	13.4
	Stopper	137	19.6
	Midfield	148	21.1
	Right Winger / Left Winger	167	23.9
	Forward	76	10.9
League	Professional Leagues	118	16.9
	U-18 and U-19	180	25.7
	U-16 and U-17	197	28.1
	U-14 and U-15	205	29.3
Number of Weekly Workouts	3 and below	180	25.7
	Between 4 and 5	385	55.0
	6 and above	135	19.3
Mental Training	Yes	456	65.1
	No	244	34.9
Providing Visual Support Training	Yes	483	69.0
	No	217	31.0
Level of Communication with Coaches	Very Good	178	25.4
	Good	176	25.1
	Moderate	147	21.0
	Bad	135	19.3
	Very Bad	64	9.1

### Data acquisition tools

In this study, a personal information form and the Football Tactical Skills Scale (FTSS) were used as data collection tools.

### Personal information form

The personal information form was developed by the researchers to record age, playing position, football background, league, weekly training frequency, mental-training status, visual-support training, and perceived communication with the coach. Visual-support training was operationalized as the player’s yes/no report of receiving visually or video-supported tactical instruction. The form did not record the specific modality, frequency, duration, or content of the visual support; therefore, this variable represents reported access to visual support rather than a standardized intervention.

### Football Tactical Skills Scale (FTSS)

The Football Tactical Skills Scale (FTSS), developed in this study, is a 17-item self-report instrument comprising four subscales: game reading and decision-making (5 items), psychological skills and game discipline (4 items), transitions in game and field utilization (5 items), and strategic thinking and risk management (3 items). Items are rated on a six-point Likert scale from 1 (strongly disagree) to 6 (strongly agree), and no items are reverse scored. Total and subscale scores are interpreted continuously, with higher scores indicating stronger self-perceived tactical skills. The FTSS does not directly observe match behaviour, test declarative tactical knowledge, or provide validated cut-off scores; consequently, its scores should not be treated as objective tactical-performance classifications or used alone for player selection decisions.

### Data analysis

SPSS 27.0 and Amos 24.0 were used for the primary analyses. Before inferential testing, missing data, univariate outliers, score distributions, and internal consistency were examined. For scale development, modified Z scores between − 3.5 and + 3.5 were accepted, and skewness and kurtosis values within ± 2 were considered compatible with approximate normality [28, 29]. In the 700-player sample, score distributions were evaluated using skewness, kurtosis, histograms, and Q–Q plots. Observations were treated as independent because each participant contributed one questionnaire record. Cronbach’s alpha, McDonald’s omega, and corrected item–total correlations were reported. Levene’s test was used to assess variance homogeneity. One-way analysis of variance (ANOVA) was used for comparisons involving three or more groups, and independent-samples t tests were used for two-group comparisons. When the homogeneity assumption was not met, Tamhane’s T2 was used for post hoc pairwise comparisons because it does not require equal variances. Effect sizes were reported as eta squared (η²) for multi-group comparisons and Cohen’s d with 95% confidence intervals for two-group comparisons. To control familywise error across the 35 primary group-comparison tests, Holm-adjusted p values were examined. Statistical significance was set at *p* < 0.05; values smaller than 0.001 are reported as *p* < 0.001. Because the design was cross-sectional, the findings are interpreted as group differences and associations rather than causal effects.

### Developing a data acquisition tool

Following standard scale-development guidance [30], an initial pool of 72 items was generated through a review of tactical skills and football-specific tactical behaviour. Evidence based on content was obtained from 10 experts: three experts in sport sciences, two football coaches, two match and performance analysts, two Turkish language and literature specialists, and one measurement and evaluation specialist. All experts held at least a master’s degree. The experts rated the relevance of each item using a four-point scale ranging from 1 (not relevant) to 4 (highly relevant). The item-level content validity index (I-CVI) was calculated as the proportion of experts rating an item as 3 or 4. Items with I-CVI values below 0.78 were considered inadequate and removed or substantially revised. With 10 experts, this criterion required agreement from at least eight experts (I-CVI = 0.80). The scale-level content validity index based on the average method (S-CVI/Ave) was calculated as the mean of the I-CVI values, with 0.90 used as the criterion for adequate scale-level content validity [31, 32]. Based on the quantitative ratings and qualitative feedback, 37 items were removed, three new items were added, and three items were reworded. The resulting 38-item trial form was resubmitted to the experts for final review. It was subsequently pilot-tested with 50 football players to assess item clarity and comprehensibility. The pilot form showed acceptable internal consistency (Cronbach’s α = 0.83), and no items were removed at this stage. Following the pilot assessment, the 38-item form was administered to an independent sample of 320 players for item analysis and exploratory factor analysis. No case exceeded the modified-Z criterion of ± 3.5, and the 38-item form showed high preliminary internal consistency (Cronbach’s α = 0.90; McDonald’s ω = 0.89). Corrected item–total correlations ranged from 0.304 to 0.598. EFA was performed using Principal Components extraction and Direct Oblimin rotation because theoretically related factors were expected. A primary loading of at least 0.30 was required. Items loading on more than one factor with a difference smaller than 0.10 between their two highest loadings were removed sequentially, and the analysis was repeated after each removal. Factor retention was guided jointly by eigenvalues greater than 1, the scree plot, theoretical interpretability, and the requirement that the final factor explain at least 5% of variance. A deliberately stringent communality criterion of 0.50 was applied. Accordingly, the psychometric evaluation began with 38 items; 21 items were removed through the item-analysis and EFA decision rules described above, yielding a 17-item, four-factor solution with communalities of 0.535–0.731 (x̄=0.600) and pattern coefficients of 0.584–0.820. This 17-item structure was subsequently evaluated and supported by CFA in an independent sample of 320 players (Table 2).

**Table 2:** KMO and Bartlett’s test of sphericity results

Kaiser–Meyer–Olkin Measure of Sampling Adequacy		0.91
Bartlett’s Test of Sphericity	χ^2^	2343.191
	df	136
	p	< 0.001

*n* = 320

*p* < 0.001

Sampling adequacy was high (KMO = 0.91), consistent with established interpretation guidelines [28, 33] Item-level measures of sampling adequacy on the diagonal of the anti-image correlation matrix ranged from 0.56 to 0.79, and all exceeded the recommended minimum threshold of 0.50, further supporting the suitability of the items for factor analysis. Bartlett’s test of sphericity was significant, χ²(136) = 2343.191, *p* < 0.001, supporting the factorability of the correlation matrix. Together with the determinant value of 0.001, these findings indicated that the item correlations were adequate for factor analysis without evidence of problematic singularity.

Principal Components extraction with Direct Oblimin rotation initially identified factors using eigenvalues greater than 1 [29]. Factor retention was not based on this criterion alone; the scree plot, theoretical coherence, the minimum 5% variance contribution of the final factor [34], item communalities, and the interpretability of the pattern matrix were considered together. Items with primary loadings below 0.30, communalities below 0.50, or cross-loadings whose two largest coefficients differed by less than 0.10 were candidates for sequential removal (Table 3).

**Table 3:** Proportion of variance explained by the factors

Variables	Eigenvalue	Variance %	Cumulative %
Factor 1	6.98	41.06	41.06
Factor 2	1.48	8.71	49.77
Factor 3	1.19	6.99	56.76
Factor 4	1.01	5.92	62.68

**Fig. 1:**
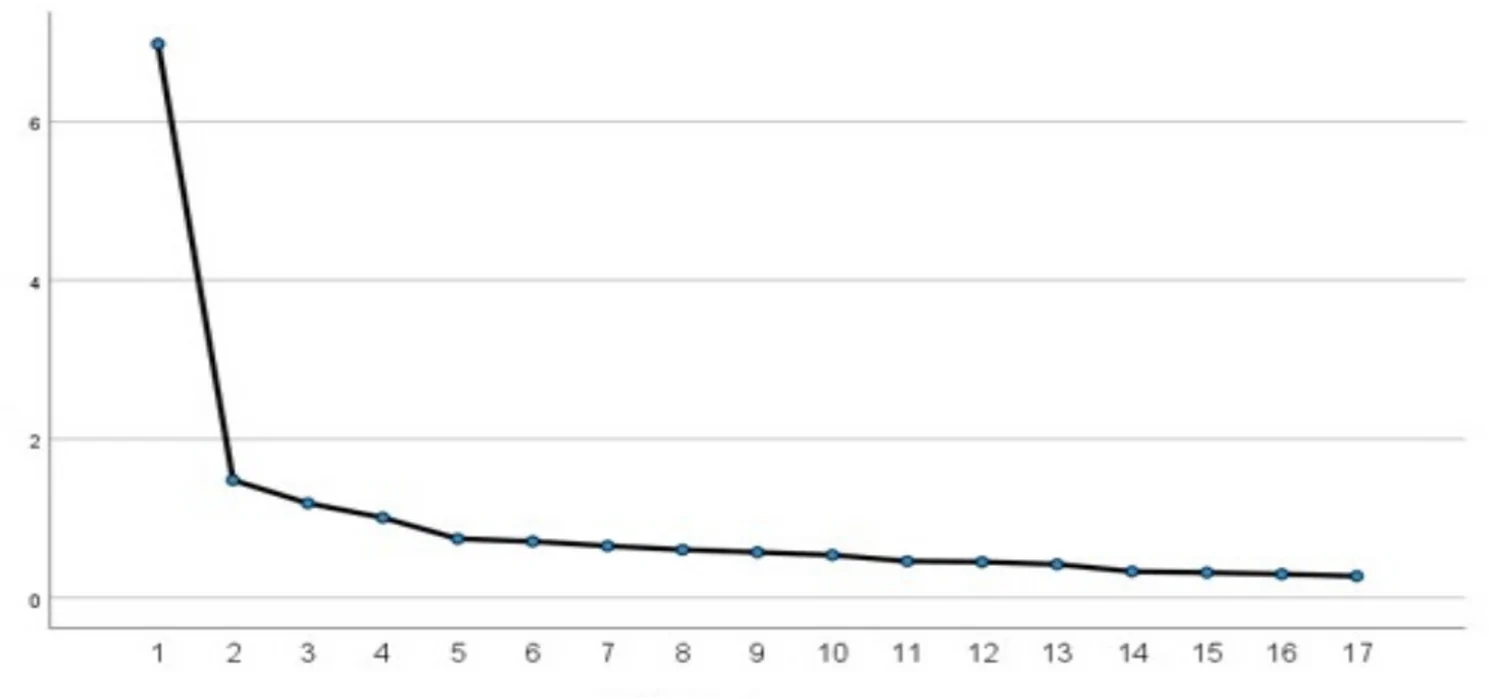
Scree plot

The final four-factor solution explained 62.68% of the total variance. The respective contributions were 41.06%, 8.71%, 6.99%, and 5.92%. All retained items met the prespecified communality criterion; communalities ranged from 0.535 to 0.731 (x̄=0.600) (Fig. 1).

The scree plot showed a marked decline followed by a clear leveling after the fourth component. This pattern, the eigenvalue criterion, theoretical interpretability, and the variance contribution of the fourth factor jointly supported retention of four factors (Table 4).

**Table 4:** Factor loading values for the scale sub-dimensions

Variables	Factor 1	Factor 2	Factor 3	Factor 4
M07	**0.754**			
M14	**0.737**			
M05	**0.704**			
M04	**0.703**			
M03	**0.679**			
M23		**0.804**		
M25		**0.749**		
M20		**0.666**		
M30		**0.664**		
M12			**0.820**	
M17			**0.719**	
M16			**0.714**	
M13			**0.615**	
M02			**0.584**	
M36				**0.772**
M37				**0.743**
M38				**0.737**

The Direct Oblimin pattern matrix showed salient coefficients of 0.679–0.754 for game reading and decision-making, 0.664–0.804 for psychological skills and game discipline, 0.584–0.820 for transitions in game and field utilization, and 0.737–0.772 for strategic thinking and risk management. Thus, all retained items exceeded the 0.30 loading criterion and met the communality and cross-loading rules described above.

**Table 5:** Comparison of football tactical skills scale by lower 27% and upper 27% groups

Variables	Group	n	x̄	sd	*t* Test		
					*t*	df	p
Football Tactical Skills Scale Total	Lower 27%	86	66.93	4.23	-36.15	170	< 0.001
	Upper 27%	86	89.35	3.90			

*p* < 0.001

An examination of Table 5 showed a statistically significant difference in Football Tactical Skills Scale total scores between the lower 27% and upper 27% groups, with the upper group scoring higher, t (170)=-36.15, *p* < 0.001. This result indicates that the scale strongly discriminated between participants with lower and higher total scores.

**Fig. 2:**
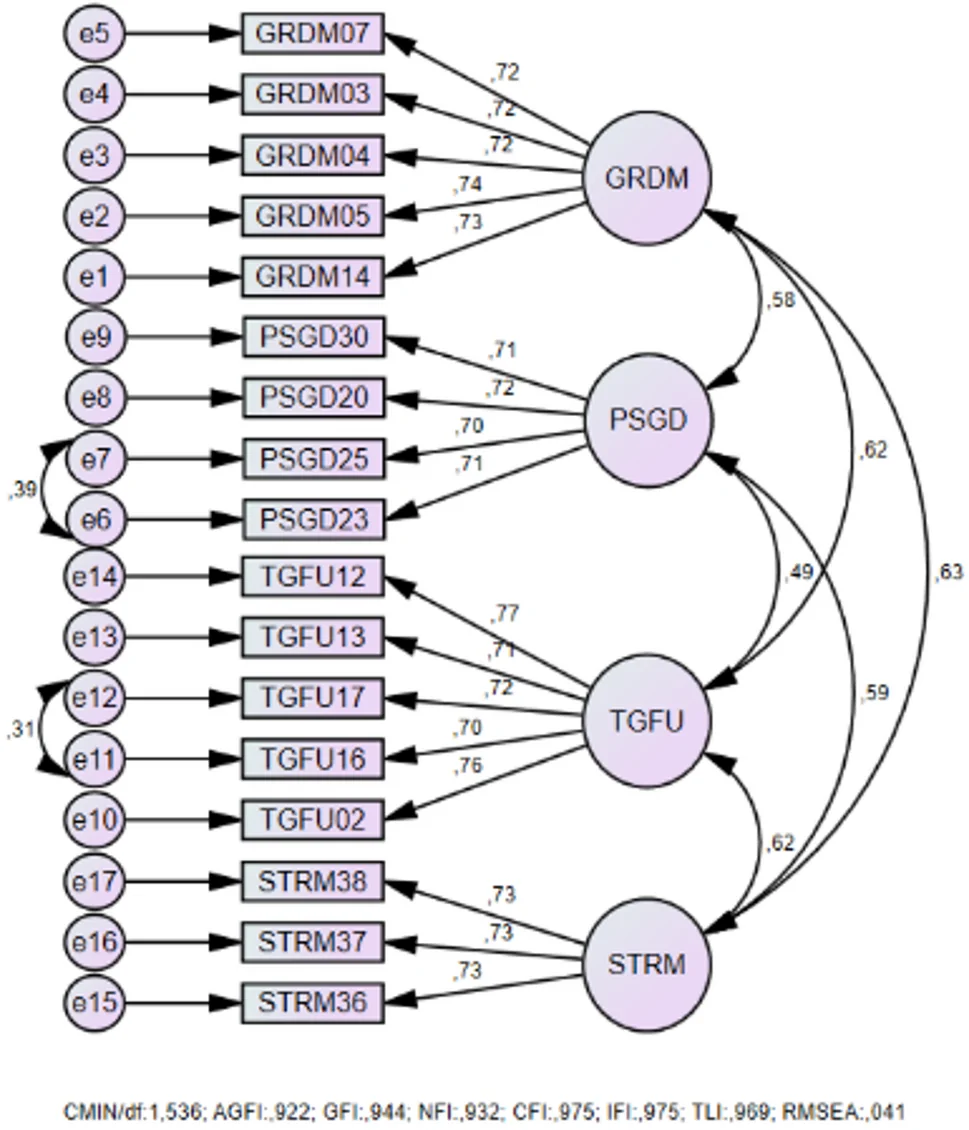
FTSS path diagram

The 17-item, four-factor structure retained after EFA was subsequently evaluated by CFA in an independent sample of 320 players. The CFA findings are shown in Fig. 2.

As shown in Fig. 2, the results of the confirmatory factor analysis indicated that the factor loadings of the game reading and decision-making sub-dimension ranged from 0.72 to 0.74, those of the psychological skills and game discipline sub-dimension ranged from 0.70 to 0.72, those of the transitions in game and field utilization sub-dimension ranged from 0.70 to 0.77, and all items of the strategic thinking and risk management sub-dimension had a factor loading of 0.73 (Table 6).

**Table 6:** Scale-specific fit index values

Fit Measure	Value	Conclusion	Perfect Fit	Acceptable Fit
χ² p-value	< 0.001	Significant; sample-size sensitive	—	—
χ²/df	1.54	Excellent	0 ≤ χ²/df ≤ 2	2 < χ²/df ≤ 3
RMSEA	0.04	Excellent	**0** ≤ RMSEA ≤ **0.05**	**0.05** ≤ RMSEA ≤ **0.08**
GFI	0.94	Acceptable	**0.95** ≤ GFI ≤ **1.00**	**0.90** ≤ GFI ≤ **0.95**
AGFI	0.92	Excellent	**0.90** ≤ AGFI ≤ **1.00**	**0.80** ≤ AGFI ≤ **0.90**
NFI	0.93	Acceptable	**0.95** ≤ NFI ≤ **1.00**	**0.90** ≤ NFI ≤ **0.95**
CFI	0.98	Excellent	**0.97** ≤ CFI ≤ **1.00**	**0.95** ≤ CFI ≤ **0.97**
TLI	0.97	Excellent	**0.97** ≤ TLI ≤ **1.00**	**0.95** ≤ TLI ≤ **0.97**

Source: [35]

The model chi-square was significant (*p* < 0.001), a result that is sensitive to sample size and was therefore not used as the sole criterion of fit. The relative chi-square (χ²/df = 1.54), RMSEA (0.04), GFI (0.94), AGFI (0.92), NFI (0.93), CFI (0.98), and TLI (0.97) collectively indicated acceptable-to-good fit of the four-factor model. Two within-factor residual covariances were retained after inspection of the modification indices because the paired items had narrowly overlapping content and parallel wording. PSGD23 and PSGD25 both concerned regulation of mental or emotional responses under adverse game conditions, whereas TGFU16 and TGFU17 were parallel items addressing the consistent application of defensive and offensive principles. These residual covariances were specified within their respective factors and did not create cross-factor paths. The findings support the internal factorial structure of the self-report instrument but do not constitute criterion validation against observed tactical performance.

Following CFA, convergent and discriminant validity were examined using composite reliability (CR), average variance extracted (AVE), maximum shared variance (MSV), average shared variance (ASV), and the Fornell–Larcker criterion [36]. Convergent validity was considered supported when CR exceeded 0.70 and AVE was approximately 0.50 or higher. Discriminant validity was evaluated by comparing AVE with MSV and ASV and by comparing the square root of AVE with interfactor correlations (Table 7).

**Table 7:** Reliability, validity, and correlation values for the factors

Factors	CR	AVE	MSV	ASV	1	2	3	4	Cronbach’s Alpha (α)	Total (α)
1	0.85	0.53	0.43	0.39	(0.73)^√AVE^				0.85	0.90
2	0.80	0.50	0.34	0.31	0.58*	(0.71)^√AVE^			0.82	
3	0.81	0.52	0.43	0.36	0.62*	0.49*	(0.72)^√AVE^		0.86	
4	0.78	0.54	0.40	0.38	0.63*	0.59*	0.62*	(0.74)^√AVE^	0.80	

*p* < 0.001

CR values ranged from 0.78 to 0.85, AVE values from 0.50 to 0.54, MSV values from 0.34 to 0.43, and ASV values from 0.31 to 0.39. For each factor, CR exceeded 0.70, AVE was at least 0.50, and AVE exceeded both MSV and ASV. The square root of AVE also exceeded the corresponding interfactor correlations. Cronbach’s alpha coefficients ranged from 0.80 to 0.86 in the CFA sample, and the total-scale alpha was 0.90. These results provide evidence for internal consistency and the convergent and discriminant validity of the four-factor measurement model; they should not be interpreted as evidence that FTSS scores are equivalent to objectively observed tactical performance.

## Results

Table 8 shows significant differences in all FTSS subscale and total scores across age, playing position, league, weekly training frequency, mental training, visual support training, and coach communication (*p* < 0.001). For the total score, eta squared was 0.229 for age, 0.208 for position, 0.229 for league, 0.115 for weekly training frequency, and 0.455 for coach communication. Mental training was associated with a large total-score difference, d = 1.31, 95% CI [1.14, 1.48], as was visual support training, d = 1.69, 95% CI [1.52, 1.87]. These results refer to self-reported FTSS scores and should not be interpreted as direct measures of observed match performance.

**Table 8:** Comparison of FTSS scores by player, training, and communication characteristics

Variables		Game Reading and Decision-Making	Psychological Skills and Game Discipline	Transitions in Game and Field Utilization	Strategic Thinking and Risk Management	FTSS Total Mean Score
		Mean ± SD	Mean ± SD	Mean ± SD	Mean ± SD	Mean ± SD
Age	Age 14–15^**(A)**^	4.61 ± 0.70	4.55 ± 0.84	4.77 ± 0.59	4.75 ± 0.72	4.67 ± 0.56
	Age 16–17 ^**(B)**^	4.82 ± 0.50	4.82 ± 0.59	4.85 ± 0.44	4.99 ± 0.50	4.86 ± 0.33
	Age 18–19^**(C)**^	5.08 ± 0.65	5.07 ± 0.60	5.10 ± 0.59	5.20 ± 0.61	5.10 ± 0.48
	Ages 20 and older^**(D)**^	5.25 ± 0.45	5.33 ± 0.53	5.18 ± 0.56	5.52 ± 0.49	5.30 ± 0.32
		F = 41.60	F = 46.16	F = 22.59	F = 53.07	F = 69.04
		*p* < 0.001	*p* < 0.001	*p* < 0.001	*p* < 0.001	*p* < 0.001
	η²	0.152	0.166	0.089	0.186	0.229
	Post Hoc	A < B - A < C - A < D - B < C - B < D - C < D				
Position	Goalkeeper^**(A)**^	4.43 ± 0.59	4.56 ± 0.66	4.62 ± 0.48	4.64 ± 0.74	4.56 ± 0.38
	Right/Left Back^**(B)**^	4.93 ± 0.54	4.91 ± 0.68	4.97 ± 0.53	5.12 ± 0.50	4.97 ± 0.41
	Stopper^**(C)**^	4.56 ± 0.69	4.52 ± 0.81	4.68 ± 0.54	4.75 ± 0.66	4.62 ± 0.53
	Midfield^**(D)**^	5.20 ± 0.68	5.09 ± 0.83	5.24 ± 0.67	5.24 ± 0.72	5.20 ± 0.58
	Right/Left Winger^**(E)**^	5.08 ± 0.49	5.07 ± 0.59	5.00 ± 0.48	5.33 ± 0.55	5.10 ± 0.35
	Forward^**(F)**^	4.93 ± 0.34	5.06 ± 0.38	4.98 ± 0.36	5.08 ± 0.40	5.01 ± 0.24
		F = 30.31	F = 16.30	F = 21.89	F = 22.44	F = 36.38
		*p* < 0.001	*p* < 0.001	*p* < 0.001	*p* < 0.001	*p* < 0.001
	η²	0.179	0.105	0.136	0.139	0.208
	Post Hoc	A < B - A < D - A < E - A < F - C < B - C < D - C < E - C < F - F < D				
League	Professional League^**(A)**^	5.32 ± 0.47	5.38 ± 0.53	5.27 ± 0.60	5.53 ± 0.52	5.36 ± 0.35
	U-18 and U-19^**(B)**^	5.08 ± 0.44	5.05 ± 0.60	5.04 ± 0.49	5.24 ± 0.53	5.09 ± 0.36
	U-16 and U-17^**(C)**^	4.76 ± 0.68	4.76 ± 0.64	4.88 ± 0.53	4.87 ± 0.67	4.81 ± 0.48
	U-14 and U-15^**(D)**^	4.62 ± 0.66	4.57 ± 0.84	4.73 ± 0.55	4.83 ± 0.66	4.68 ± 0.52
		F = 44.33	F = 41.06	F = 27.74	F = 44.12	F = 68.99
		*p* < 0.001	*p* < 0.001	*p* < 0.001	*p* < 0.001	*p* < 0.001
	η²	0.160	0.150	0.107	0.160	0.229
	Post Hoc	A < B - A < C - A < D - B < C - B < D				
Number of Weekly Workouts	3 and below^**(A)**^	4.61 ± 0.71	4.51 ± 0.86	4.78 ± 0.58	4.78 ± 0.58	4.67 ± 0.57
	Between 4 and 5^**(B)**^	4.93 ± 0.52	4.97 ± 0.56	4.93 ± 0.47	4.93 ± 0.47	4.98 ± 0.36
	6 and above^**(C)**^	5.16 ± 0.71	5.15 ± 0.81	5.20 ± 0.72	5.20 ± 0.72	5.18 ± 0.62
		F = 32.66	F = 38.98	F = 21.97	F = 18.18	F = 45.27
		*p* < 0.001	*p* < 0.001	*p* < 0.001	*p* < 0.001	*p* < 0.001
	η²	0.086	0.101	0.059	0.050	0.115
	Post Hoc	A < B - A < C - B < C				
Mental Training	Yes	5.11 ± 0.54	5.09 ± 0.64	5.10 ± 0.54	5.30 ± 0.54	5.14 ± 0.41
	No	4.50 ± 0.62	4.50 ± 0.74	4.65 ± 0.51	4.63 ± 0.65	4.57 ± 0.45
		t = 12.83	t = 10.50	t = 10.77	t = 13.56	t = 16.17
		*p* < 0.001	*p* < 0.001	*p* < 0.001	*p* < 0.001	*p* < 0.001
	Cohen’s d (95% CI)	1.06 [0.90, 1.23]	0.87 [0.71, 1.03]	0.84 [0.68, 1.00]	1.13 [0.97, 1.30]	1.31 [1.14, 1.48]
Providing Visual Support Training	Yes	5.18 ± 0.50	5.13 ± 0.59	5.17 ± 0.48	5.46 ± 0.42	5.22 ± 0.36
	No	4.49 ± 0.60	4.55 ± 0.78	4.63 ± 0.53	4.52 ± 0.55	4.55 ± 0.42
		t = 16.04	t = 10.78	t = 13.52	t = 24.18	t = 21.60
		*p* < 0.001	*p* < 0.001	*p* < 0.001	*p* < 0.001	*p* < 0.001
	Cohen’s d (95% CI)	1.26 [1.10, 1.43]	0.86 [0.70, 1.02]	1.05 [0.89, 1.21]	1.93 [1.75, 2.11]	1.69 [1.52, 1.87]
Level of Communication with Coaches	Very Good^**(A)**^	5.34 ± 0.50	5.35 ± 0.56	5.34 ± 0.60	5.38 ± 0.59	5.35 ± 0.41
	Good^**(B)**^	5.06 ± 0.38	5.05 ± 0.51	4.95 ± 0.34	5.29 ± 0.46	5.06 ± 0.22
	Moderate^**(C)**^	4.82 ± 0.56	4.80 ± 0.66	4.86 ± 0.47	5.07 ± 0.56	4.87 ± 0.39
	Bad^**(D)**^	4.58 ± 0.58	4.61 ± 0.74	4.76 ± 0.57	4.69 ± 0.63	4.66±0.47
	Very Bad^**(E)**^	4.02 ± 0.60	3.95 ± 0.67	4.36 ± 0.46	4.35 ± 0.70	4.16 ± 0.33
		F = 96.49	F = 70.52	F = 55.43	F = 58.32	F = 145.09
		*p* < 0.001	*p* < 0.001	*p* < 0.001	*p* < 0.001	*p* < 0.001
	η²	0.357	0.289	0.242	0.251	0.455
	Post Hoc	A > B - A > C - A > D - A > E - B > C - B > D - B > E - C > E - D > E				

All reported group comparisons were p < 0.001 and remained significant after Holm correction across 35 primary group-comparison tests

*n* = 700

F = one-way ANOVA; Tamhane’s T2 was used for post hoc pairwise comparisons when Levene’s test indicated nonhomogeneous variances

t=independent-samples t test

η²=eta squared

d=Cohen’s d with 95% CI

No item-level data were missing in the 700-player dataset. Subscale and total-score skewness values ranged from − 0.735 to − 0.210, and kurtosis values ranged from 0.318 to 0.642, supporting approximate univariate normality for the planned large-sample analyses. Internal consistency was acceptable to good for game reading and decision-making (α = 0.793, ω = 0.793), psychological skills and game discipline (α = 0.747, ω = 0.750), transitions in game and field utilization (α = 0.790, ω = 0.794), and strategic thinking and risk management (α = 0.736, ω = 0.738), and was good for the total score (α = 0.883, ω = 0.886). Corrected item–total correlations ranged from 0.423 to 0.574.

Levene’s test was significant for the FTSS total score across age (F = 29.68), position (F = 24.82), league (F = 7.11), weekly training frequency (F = 81.11), and coach communication (F = 17.88), all *p* < 0.001. One-way ANOVA showed significant total-score differences across age, position, league, weekly training frequency, and coach communication (all *p* < 0.001); because variance homogeneity was not supported, Tamhane’s T2 was used for post hoc pairwise comparisons. Across the complete set of 35 subscale and total-score group tests, all Holm-adjusted p values remained below 0.001. Independent-samples t tests were used for the two-group comparisons, and effect sizes were accompanied by 95% confidence intervals.

## Discussion

The present study had two complementary purposes: to develop and initially validate the Football Tactical Skills Scale (FTSS) and to examine differences in FTSS scores across selected player, training, and psychosocial characteristics. The analyses supported a 17-item, four-factor structure comprising game reading and decision-making, psychological skills and game discipline, transitions in game and field utilization, and strategic thinking and risk management. The independent exploratory and confirmatory samples, satisfactory model fit, internal-consistency coefficients, lower-upper group discrimination, and convergent and discriminant validity indicators provide initial evidence that the FTSS can be used to obtain a structured profile of football players’ self-reported tactical skills. The multidimensional structure is also theoretically coherent because tactical action requires players to perceive and interpret game information, regulate attention and behaviour under pressure, coordinate offensive and defensive transitions, use space, and evaluate strategic risk. Nevertheless, these psychometric findings establish the internal functioning of the questionnaire rather than equivalence with objectively observed tactical performance.

This distinction is central to the interpretation of all comparative findings. Previous self-report research has shown that players’ tactical self-assessments can distinguish developmental and competitive groups and can provide information about perceived positioning and decision-making [1, 15, 16]. However, verbal self-reports and online tactical behaviour do not necessarily correspond closely [17]. Accordingly, the group differences observed in the present study should be interpreted as differences in perceived tactical functioning. They provide preliminary known-groups sensitivity for the FTSS, but they do not demonstrate objective tactical superiority or causal effects of the examined characteristics.

### Interpretation of the FTSS factor structure

Beyond the statistical support for the four-factor structure, the content of the retained items provides a theoretically meaningful representation of players’ perceived tactical functioning. The four dimensions reflect related but distinguishable aspects of how players evaluate their own decision-making, game-specific self-regulation, spatial and transition-related actions, and strategic risk assessment. Their moderate intercorrelations are consistent with the view that tactical functioning emerges from the coordinated operation of perceptual-cognitive, psychological, spatial, and strategic processes rather than from a single isolated ability. Nevertheless, because the FTSS is a self-report questionnaire, the following interpretations concern the constructs represented by the item content and players’ perceptions of their own functioning; they do not demonstrate objectively observed tactical behaviour.

### Game reading and decision-making

The first factor, game reading and decision-making, represents players’ perceived capacity to interpret changing game situations, identify contextually appropriate options, coordinate their actions with teammates, and make decisions under pressure. Game reading depends on the detection of relevant environmental information, recognition of emerging patterns, anticipation of possible developments, and selection of an action that is compatible with team principles and situational constraints. Previous research has shown that players selected for professional academies may demonstrate more advanced game-reading abilities than non-selected players and that experience and expertise are associated with more effective decision-making in dynamic sporting contexts [37, 38]. Football decision-making also requires cognitive judgements to be coupled with timely motor responses under spatial and temporal pressure [6, 39, 40].

The inclusion of passing decisions, coordination, adherence to the team’s tactical understanding, decision-making under pressure, and overall game reading within the same factor is therefore theoretically coherent. These items collectively reflect how players evaluate their ability to recognise relevant information and translate it into tactically appropriate action. However, a high score on this dimension indicates stronger perceived competence rather than demonstrating that the player consistently selects the most effective option during actual match play. Future studies should compare this subscale with video-based decision tasks, coach ratings, and observational indicators of scanning, positioning, and response selection.

### Psychological skills and game discipline

The second factor, psychological skills and game discipline, reflects players’ perceptions of their ability to maintain concentration, emotional control, composure, and confidence during competitive situations. These characteristics may support tactical functioning by helping players preserve role responsibilities and implement tactical intentions despite pressure, mistakes, fatigue, opposition behaviour, or rapidly changing game conditions. Concentration and emotional regulation are particularly relevant because successful tactical action often requires players to inhibit unsuitable responses, remain attentive to evolving information, and continue following collective principles when under stress [41–43]. Mental toughness and self-efficacy may similarly contribute to sustained focus and confidence during demanding periods of play [44].

This factor should not be interpreted as suggesting that general psychological resilience is equivalent to tactical expertise. Rather, the retained items represent game-specific self-regulatory resources that may support the consistent execution of tactical responsibilities. Discipline in this context refers to maintaining concentration, emotional stability, confidence, and commitment to collective roles during play. The dimension therefore captures players’ self-perceived capacity to regulate their psychological responses in ways that support tactical action. Its distinction from broader psychological constructs should be examined in future research using measures of mental toughness, emotional regulation, anxiety, and general self-efficacy.

### Transitions in game and field utilization

The third factor, transitions in game and field utilization, represents players’ perceptions of their ability to use width and depth, adopt advantageous positions, apply offensive and defensive principles, and adapt their actions following changes in possession. Tactical functioning in football requires continuous reorganisation as teams move between attacking and defensive phases. The use of space is influenced by formation, position-specific responsibilities, teammates’ movements, opposition behaviour, and the location of the ball [21, 22]. Appropriate width and depth may create passing options and stretch defensive structures, while rapid positional adjustment during transitions may support defensive recovery or exploitation of temporary attacking space.

The grouping of offensive principles, defensive principles, width, depth, and positioning within a single factor is consistent with the interdependence of spatial organisation and phase transitions. Players must not only recognise available space but also understand how their position contributes to collective structure before and after possession changes. Previous work has similarly emphasised the importance of spatial occupation, coordinated movement, and balanced offensive-defensive organisation in football performance [45–47]. Nevertheless, the FTSS does not directly quantify players’ coordinates, movement synchronisation, or transition speed. Higher scores indicate that players perceive themselves as more competent in spatial and transition-related actions; future validation should compare this dimension with positional tracking, notational analysis, and representative small-sided games.

### Strategic thinking and risk management

The fourth factor, strategic thinking and risk management, reflects players’ perceived ability to adjust their decisions according to the score, determine when risk-taking may be appropriate, and consider the strengths and weaknesses of the opposition. These items concern the broader strategic context of play rather than only the immediate execution of an isolated action. Football situations require players to evaluate potential benefits and costs while accounting for match status, available time, tactical objectives, opposition characteristics, and the consequences of losing possession or committing players forward. Strategic understanding may therefore shape how players frame game problems and select solutions when technical or physical options alone are insufficient [7, 40].

Although this factor contains only three items, they form a conceptually coherent domain centred on contextual judgement, tactical foresight, and risk evaluation. The factor differs from basic decision-making because it concerns how decisions are modified in response to broader match conditions. A player may identify a technically possible action yet reject it because the score, time remaining, or opposition structure makes the risk inappropriate. Conversely, the same action may become justified when the team needs to create an immediate scoring opportunity. Higher scores therefore represent stronger self-perceived strategic and risk-management competence, not evidence that the player’s match decisions were objectively optimal. Future studies should assess this dimension through scenario-based tests and match observations in which decision quality can be evaluated relative to the tactical context.

### Age and developmental context

FTSS total and subscale scores differed across age groups, with progressively higher mean scores in older groups and a large effect for the total score (η²=0.229). Research supporting this pattern includes studies of tactical knowledge, decision-making, cognitive performance, and game behaviour in youth football [48–53]. Bairachnyi [48], reported age-related differences in cognitive abilities among U11-U17 football players, whereas Heilmann et al. [51] found that older youth players performed better on football-related cognitive tests. In game-based research, Figueira et al. [50] and Machado et al. [53] also showed that age category and tactical proficiency were associated with differences in positional and collective tactical behaviour.

Several mechanisms may contribute to this pattern. As players advance through developmental stages, tactical instruction generally shifts from basic individual and group principles toward more complex team organisation, competition tactics, and position-specific responsibilities [54, 55]. Repeated exposure to varied game states may strengthen cue recognition, anticipation, the retrieval of tactical solutions, and the ability to coordinate decisions with teammates. Greater football experience may also support emotional control and confidence when responding to pressure. These processes are compatible with the higher scores observed across all four FTSS dimensions, rather than only in game reading and decision-making.

Age should not, however, be interpreted as an isolated developmental cause. In the present sample, older players were also more likely to have accumulated more years of training, competed at higher levels, and experienced more demanding coaching environments. Biological maturation, selection into stronger teams, confidence, and familiarity with tactical terminology may also influence self-ratings. Some previous work has not identified age-related differences in self-reported tactical skills [56], indicating that age effects may depend on the age range, competitive context, measurement approach, and distribution of experience. Longitudinal studies are therefore required to separate maturation from accumulated practice and selection. In applied use, age-group profiles should support developmentally appropriate reflection and observation rather than be treated as fixed normative cut-offs.

### Playing position, physical demands, and tactical roles

Playing position was associated with all FTSS dimensions and the total score, with a large total-score effect (η²=0.208). Midfielders reported the highest total, game-reading, and transition-related scores, whereas wide players reported the highest strategic thinking and risk-management scores. Similar positional differences have been reported in studies of tactical knowledge, decision-making, and small-sided-game behaviour [56–58]. This literature supports the interpretation that the tactical problems repeatedly encountered by players differ according to role, field location, and the phase of play in which they are most frequently involved.

The midfield finding is particularly consistent with the functional demands of the position. Midfielders connect defensive and attacking lines, receive information from multiple directions, support ball circulation, manage tempo, and participate repeatedly in both offensive and defensive transitions [52, 59]. Arjol-Serrano et al. [60] found that midfielders performed a broad range of technical-tactical actions across formations, including more passing and field-involvement actions. Forcher et al. [61] likewise showed that formation and playing position jointly shape technical and physical match demands. These responsibilities require frequent scanning before receiving the ball, rapid updating of spatial information, and continuous adjustment to teammates’ and opponents’ movement, which may strengthen both tactical self-awareness and the perception of competence.

Tactical behaviour should also be considered together with physical match demands. Recent GPS research in elite U19 football found that central midfielders covered the greatest total distance and that wide positions performed substantial high-speed work, while central defenders displayed lower high-speed demands because of their tactical role and operating area [62, 63]. Earlier studies similarly reported positional differences in total distance, accelerations, decelerations, and high-intensity running [64]. These findings do not mean that running output is equivalent to tactical quality. Rather, physical capacity, available space, formation, and transition speed constrain which tactical options can be reached and executed. The high strategic thinking and risk-management scores of wide players may reflect repeated decisions about when to advance, attack space, engage in one-to-one actions, cross, recover, or accept the defensive risk created by forward movement. Their speed and agility profiles may facilitate these role demands [59, 65].

Goalkeepers obtained the lowest mean FTSS scores. This result must not be interpreted as evidence that goalkeepers possess inferior tactical ability. Goalkeeping involves specialised perceptual, positioning, distribution, communication, and penalty-area decision demands that differ substantially from those of outfield players [66]. Several FTSS items refer to width, depth, offensive and defensive principles, and field positioning in ways that may be experienced differently by goalkeepers. The observed difference may therefore reflect genuine role specificity, unequal exposure to the behaviours represented in the scale, or differences in item relevance. Because measurement invariance across positions was not tested, direct rank-order interpretations between goalkeepers and outfield players should be made cautiously. Future validation should examine goalkeeper-specific functioning and whether selected items require role-sensitive interpretation.

### Competitive level and weekly training exposure

Players competing at higher league levels reported higher FTSS scores, and the total-score effect was large (η²=0.229). Supporting evidence is available from studies comparing tactical knowledge and decision-making across expertise or competition levels [38, 67–70]. Kannekens et al. [16], found differences in self-assessed tactical skills between high-level youth teams, while Del-Campo et al. [38] reported more effective decision-making among experienced than novice invasion-game players. Keller et al. [68] also showed that football-specific tactical decision measures could discriminate between youth skill levels.

Higher competitive levels may expose players to faster game tempos, more compact spaces, stronger opposition, more detailed tactical preparation, and greater accountability for role execution. Such environments can create richer learning opportunities and may improve players’ tactical vocabulary and confidence in evaluating their own actions. At the same time, league differences can arise from selection: players who already possess stronger perceptual-cognitive, technical, physical, or psychological characteristics are more likely to enter and remain in higher-level teams. Club resources, coaching quality, match exposure, age, and training volume may also differ systematically between leagues. The cross-sectional design cannot distinguish development produced by the competitive environment from prior characteristics that influenced selection. The league findings should therefore be interpreted as group associations rather than evidence that progression to a higher league causes improvement in tactical skills.

Weekly training frequency was also associated with FTSS scores, with a moderate-to-large effect for the total score (η²=0.115). Studies supporting a role for structured practice include work on high-quality tactical-development activities, task difficulty, and periodised technical-tactical training [53, 67, 71, 72]. Repeated practice may increase encounters with representative tactical problems, provide more opportunities for feedback, and facilitate perceptual learning, pattern recognition, and tactical automatisms. When practice preserves the informational and action constraints of competition, players can learn to couple perception with context-appropriate movement rather than memorise decontextualised solutions [12].

Training frequency alone is nevertheless an incomplete indicator of learning quality. The study did not record session duration, intensity, coaching approach, task representativeness, tactical content, or accumulated training age. More sessions may also identify players in higher-level programmes with better facilities and stronger support systems. Elek [73] did not find a comparable association, suggesting that the relationship may depend more on the content and quality of practice than on frequency itself. Coaches should therefore avoid interpreting the present result as a simple dose-response rule. The finding supports careful examination of how often players encounter meaningful tactical tasks, but experimental and longitudinal work is needed to determine which practice designs produce changes in perceived and observed tactical performance.

### Mental training and psychological regulation

Players who reported engaging in mental training had higher FTSS scores across all dimensions, with a large total-score difference (d = 1.31, 95% CI [1.14, 1.48]). This pattern is consistent with literature linking psychological preparation, self-regulation, mental toughness, mindfulness, and perceptual-cognitive training with attention, anticipation, decision-making, and sport performance [74–78]. Zhu et al. [79], in a systematic review and meta-analysis, concluded that perceptual-cognitive training can improve anticipation and decision-making in team-sport contexts. These capacities are directly relevant to the FTSS domains because players must sustain attention, inhibit unsuitable responses, update information, regulate emotional reactions, and select actions under time pressure.

Evidence concerning mental fatigue provides a complementary explanation. Kunrath et al. [80] showed that mental fatigue altered cognitive, tactical, and physical responses during football small-sided games. More recent professional-football evidence indicated marked post-match increases in perceived mental fatigue and deterioration in reaction time, accuracy, processing speed, and response consistency [81]. Mental preparation may help players recognise and manage attentional disruption, perceived pressure, and emotional responses, thereby supporting confidence in tactical functioning. However, the current mental-training item recorded only whether players reported such training; it did not identify the method, frequency, duration, provider, or adherence. Players who seek mental training may also differ in motivation, competitive level, club support, and self-efficacy. The observed difference therefore does not establish that mental training caused higher tactical skill. Future studies should evaluate clearly specified programmes and include behavioural decision measures alongside FTSS scores.

### Visual support and perceptual-cognitive learning

The largest two-group difference was observed for visual support training (FTSS total d = 1.69, 95% CI [1.52, 1.87]). Research supporting visual and video-based approaches includes football-specific work on anticipation and decision-making and broader team-sport evidence on decision-training programmes [69, 70]. Zhao et al. [82] concluded that video-based training can support anticipation and decision-making in football, while Silva et al. [70] reported beneficial effects of decision-making training programmes in youth team-sport players. Video and other visual representations allow players to revisit critical moments, compare tactical alternatives, and discuss cues that may be missed during live play.

Potential mechanisms include improved visual search, allocation of gaze to informative locations, peripheral awareness, pattern recognition, anticipation, and pre-reception scanning. Visual feedback may also slow down or replay complex sequences, allowing the player and coach to connect an observed cue with the action selected and its consequence. These processes are compatible with the higher scores observed in game reading, transitions, and strategic decision-making. Nevertheless, the study did not evaluate a standardised visual intervention. Visual support was measured as a yes/no self-report, and information about whether it involved match video, individual clips, animation, tactical boards, virtual simulation, frequency, duration, or instructional quality was not collected. The result should therefore be described as an association with reported exposure to visual support, not as evidence for the efficacy of a particular video or technology-based programme. The large effect size may also reflect differences in club resources, competition level, and coaching structure.

### Coach-player communication and the learning environment

Perceived communication quality with coaches showed the largest multigroup effect on the FTSS total score (η²=0.455), with progressively higher scores across better communication categories. Studies of the coach-athlete relationship, motivational climate, social support, leadership, and cognitive or psychophysiological functioning provide indirect support for this association [83–88]. Zhang and Rhim [89], reported links between coach-athlete relationship quality, psychological needs, and motor behaviour, whereas Davis et al. [83] connected relationship quality with cognitive functioning and exhaustion in team-sport athletes.

In tactical learning, communication is valuable when it makes game information clearer without removing the player’s need to perceive and solve problems. Specific, timely feedback can help players understand why a decision was effective, identify cues that were overlooked, and connect individual actions with collective principles. Guided questions and representative tasks may encourage players to explore solutions and develop shared tactical understanding rather than depend on prescriptive instructions. This interpretation is consistent with representative learning design, which emphasises practice environments that preserve the information-action relations of competition [12]. Effective communication may also create psychological safety for players to ask questions, acknowledge errors, and reflect more accurately on their tactical choices.

The magnitude of the association requires caution. Both coach communication and tactical skills were reported by the same player at the same time. A generally positive view of the coach or team environment may have influenced responses to both variables, producing common-method or halo effects. Reverse direction is also possible: players who feel tactically competent, receive more playing time, or are more engaged may communicate more frequently and evaluate that communication more positively. Communication quality was assessed with a single broad item rather than a validated multidimensional relationship measure. The finding identifies a potentially important learning-context variable, but it does not demonstrate that improving communication alone will produce the observed score differences.

### Contribution and practical applications of the FTSS

The principal contribution of the study is the development of a concise football-specific questionnaire that provides four related self-perception profiles and can be administered to large and heterogeneous groups. The same instrument identified systematic differences across age, position, competition level, training exposure, mental training, visual support, and coach communication. This pattern supports preliminary sensitivity to theoretically relevant group characteristics, while the effect-size estimates indicate that the magnitude of differences was not uniform across variables. The FTSS may therefore help researchers and practitioners move beyond a single total score and identify domains that warrant closer examination.

In practice, the FTSS should be used as a complement to observation rather than as a substitute for it. A player reporting relatively low game-reading scores but adequate transition scores could be observed specifically for scanning, positioning before receiving, and response selection under pressure. A low psychological-skills and game-discipline profile could prompt discussion about concentration, emotional regulation, and adherence to collective responsibilities. Disagreement between a player’s profile and coach observation may itself be useful by identifying overconfidence, underconfidence, unclear role expectations, or communication gaps. Repeated administration may support structured reflection, although meaningful interpretation of change requires future evidence on temporal stability and responsiveness.

The FTSS should not be used alone for talent identification, selection, deselection, position assignment, or claims about objective match performance. No validated cut-off scores are available, and the present data do not justify labels such as low, moderate, high, competent, or incompetent. Continuous subscale profiles and within-player change are more defensible than categorical classification. Where possible, FTSS findings should be triangulated with FUT-SAT, GPAI, coach or expert ratings, notational analysis, positional data, video-based decision tasks, and representative small-sided games [10, 11].

### Limitations and future research

Several limitations define the boundaries of the findings. The FTSS and all exposure variables were self-reported. Social desirability, confidence, response style, limited self-awareness, and common-method variance may have inflated the observed associations. Criterion validity was not examined against observed tactical behaviour, coach ratings, expert assessment, FUT-SAT, GPAI, notational analysis, positional data, or representative game tasks. Consequently, the study supports the psychometric functioning of a questionnaire measuring perceived tactical skills, but not the accuracy of those perceptions or their correspondence with match performance.

The comparative component was cross-sectional. Age, league level, training frequency, mental training, visual support, and coach communication are likely to overlap, while unmeasured factors such as accumulated training age, biological maturation, playing time, club resources, coaching philosophy, tactical formation, and prior success may also have contributed to the group differences. The analyses were designed as unadjusted group comparisons; therefore, causal direction and independent effects cannot be inferred. Longitudinal research should follow players across developmental transitions, and experimental studies should evaluate specified mental, visual, communication, and representative-practice interventions.

Mental training and visual support were measured using broad yes/no items, whereas coach communication was assessed through a single perceived-quality item. Future studies should record modality, content, dose, duration, provider, adherence, and instructional context and should use validated multidimensional measures of the coach-athlete relationship. In addition, internal consistency does not establish temporal stability. Test-retest reliability, intraclass correlation coefficients, measurement error, responsiveness, and minimal detectable change should be examined before the FTSS is used to monitor individual change.

Measurement invariance was not tested across age, position, league, or other subgroups. Group mean differences cannot be interpreted confidently as differences in the same latent construct until configural, metric, and scalar invariance are evaluated. This issue is particularly relevant for goalkeepers because some outfield-oriented tactical items may function differently for that role. The samples were also drawn from football players in Türkiye. Cross-cultural adaptation and independent replication are therefore required before generalising the factor structure and score interpretation to other football cultures, languages, genders, and competitive systems.

## Conclusion

The study provides initial support for the FTSS as a 17-item, four-dimensional self-report questionnaire assessing players’ perceived tactical skills in football. Its factor structure, internal consistency, lower-upper group discrimination, and convergent and discriminant validity indicators were satisfactory in the development samples, while reliability was also supported in the 700-player comparison sample.

Higher FTSS scores were observed among older players, midfielders and wide players in role-specific domains, players competing at higher levels, players reporting more weekly training, mental training, visual support, and better coach communication. These findings indicate systematic differences in self-perceived tactical functioning, not causal effects or direct evidence of observed match behaviour. The FTSS is best positioned as a profile-based reflective tool used alongside observational and representative assessments.

Future research should establish criterion validity, test-retest reliability, responsiveness, and measurement invariance; evaluate the scale across countries, genders, positions, and competition levels; and use longitudinal or experimental designs to examine how specific training and communication practices relate to changes in both perceived and objectively observed tactical performance.

## Supplementary Information

Supplementary Material 1.

## Data Availability

The datasets used and/or analysed during the current study are available from the corresponding author on reasonable request.

## Declarations

### Author Note

This article was derived from the doctoral dissertation of Oğuzhan Eroğlu.

## Abbreviations

FTSS: Football Tactical Skills Scale
EFA: Exploratory factor analysis
CFA: Confirmatory factor analysis
KMO: Kaiser–Meyer–Olkin
ASV: Average Shared Variance
MSV: Maximum Shared Variance
AVE: Average Variance Extracted
CR: Composite Reliability
SD: Standard Deviation
ANOVA: Analysis of Variance
GRDM: Game Reading and Decision-Making
PSGD: Psychological Skills and Game Discipline
TGFU: Transitions in Game and Field Utilization
STRM: Strategic Thinking and Risk Management
