# Supplementary material for: Development of the football tactical skills scale and comparison of scores across player and training characteristics
Source: BMC Sports Sci Med Rehabil. 2026 Aug 1;18:532. doi: 10.1186/s13102-026-01934-z (PMC13721684; doi:10.1186/s13102-026-01934-z)
Supplement: Supplementary file 1 [file 13102_2026_1934_MOESM1_ESM.docx]

**Supplementary Information**

**Development of the Football Tactical Skills Scale and Comparison of Scores Across Player and Training Characteristics**

**Supplementary Appendix 1**

**Football Tactical Skills Scale (FTSS) – English Version**

|  | Strongly disagree | Disagree | Slightly disagree | Slightly agree | Agree | Strongly agree |
| --- | --- | --- | --- | --- | --- | --- |
| **Game Reading and Decision-Making Sub-Dimension** | | | | | | |
| 1. I believe I am successful in playing the ball to my teammates at the right time. | 1 | 2 | 3 | 4 | 5 | 6 |
| 2. I believe I’m good at making the right decisions when I’m under pressure. | 1 | 2 | 3 | 4 | 5 | 6 |
| 3. I believe I am successful in making the most appropriate decisions during a game by adhering to my team’s tactical understanding. | 1 | 2 | 3 | 4 | 5 | 6 |
| 4. During the game, I can work in coordination with my teammates. | 1 | 2 | 3 | 4 | 5 | 6 |
| 5. I believe I am successful in accurately reading the game. | 1 | 2 | 3 | 4 | 5 | 6 |
| **Psychological Skills and Game Discipline Sub-Dimension** | | | | | | |
| 6. I believe I am successful in not letting external factors negatively affect my mental state. | 1 | 2 | 3 | 4 | 5 | 6 |
| 7. I believe I am able to stay calm when I encounter a difficult situation during the game. | 1 | 2 | 3 | 4 | 5 | 6 |
| 8. I believe I am successful in maintaining a high level of concentration during a match. | 1 | 2 | 3 | 4 | 5 | 6 |
| 9. I believe I am successful in maintaining a high level of self-confidence during a match. | 1 | 2 | 3 | 4 | 5 | 6 |
| **Transitions in Game and Field Utilization Sub-Dimension** | | | | | | |
| 10. I believe I am successful in adding depth to the game. | 1 | 2 | 3 | 4 | 5 | 6 |
| 11. I believe I am successful in adding width to the game. | 1 | 2 | 3 | 4 | 5 | 6 |
| 12. I believe I do a good job of applying defensive principles correctly throughout the game. | 1 | 2 | 3 | 4 | 5 | 6 |
| 13. I believe I do a good job of correctly applying our offensive principles throughout the game. | 1 | 2 | 3 | 4 | 5 | 6 |
| 14. I can gain an advantage over my opponents by taking correct positions during the game. | 1 | 2 | 3 | 4 | 5 | 6 |
| **Strategic Thinking and Risk Management Sub-Dimension** | | | | | | |
| 15. I believe I am successful in managing phases of play appropriately according to the score during a game. | 1 | 2 | 3 | 4 | 5 | 6 |
| 16. I believe I am successful in knowing when to take risks during a game. | 1 | 2 | 3 | 4 | 5 | 6 |
| 17. I believe I am successful in making decisions during the game based on the opponent’s strengths and weaknesses. | 1 | 2 | 3 | 4 | 5 | 6 |

**Supplementary Appendix 2**

**Futbolda Taktiksel Beceriler Ölçeği (FTBÖ) – Turkish Version**

|  | Kesinlikle Katılmıyorum | Katılmıyorum | Kısmen Katılmıyorum | Kısmen Katılıyorum | Katılıyorum | Kesinlikle Katılıyorum |
| --- | --- | --- | --- | --- | --- | --- |
| **Oyunu Okuma ve Karar Verme Alt Boyutu** | | | | | | |
| 1. Topu, arkadaşlarımla en doğru zamanda buluşturabilme konusunda başarılı olduğumu düşünüyorum. | 1 | 2 | 3 | 4 | 5 | 6 |
| 2. Baskı altında kaldığımda doğru karar verebilme konusunda başarılı olduğumu düşünüyorum. | 1 | 2 | 3 | 4 | 5 | 6 |
| 3. Maç süresince, takımımın taktiksel anlayışına bağlı kalarak en doğru kararı verme konusunda başarılı olduğumu düşünüyorum. | 1 | 2 | 3 | 4 | 5 | 6 |
| 4. Maç süresince, takım arkadaşlarım ile koordineli bir şekilde hareket edebilirim. | 1 | 2 | 3 | 4 | 5 | 6 |
| 5. Oyunu doğru okuyabilme konusunda başarılı olduğumu düşünüyorum. | 1 | 2 | 3 | 4 | 5 | 6 |
| **Psikolojik Beceriler ve Oyun Disiplini Alt Boyutu** | | | | | | |
| 6. Dış faktörlerin psikolojimi olumsuz yönde etkilemesine izin vermeme konusunda başarılı olduğumu düşünüyorum. | 1 | 2 | 3 | 4 | 5 | 6 |
| 7. Maç süresince olumsuz bir durumla karşılaştığımda sakin kalma konusunda başarılı olduğumu düşünüyorum. | 1 | 2 | 3 | 4 | 5 | 6 |
| 8. Maç konsantrasyonumu yüksek seviyede tutabilme konusunda başarılı olduğumu düşünüyorum. | 1 | 2 | 3 | 4 | 5 | 6 |
| 9. Maç süresince öz güvenimi yüksek seviyede tutabilme konusunda başarılı olduğumu düşünüyorum. | 1 | 2 | 3 | 4 | 5 | 6 |
| **Oyun Geçişleri ve Alan Kullanımı** **Alt Boyutu** | | | | | | |
| 10. Oyuna derinlik kazandırma konusunda başarılı olduğumu düşünüyorum. | 1 | 2 | 3 | 4 | 5 | 6 |
| 11. Oyuna genişlik kazandırma konusunda başarılı olduğumu düşünüyorum. | 1 | 2 | 3 | 4 | 5 | 6 |
| 12. Maç süresince, savunma prensiplerini doğru bir şekilde uygulama konusunda başarılı olduğumu düşünüyorum. | 1 | 2 | 3 | 4 | 5 | 6 |
| 13. Maç süresince, hücum prensiplerini doğru bir şekilde uygulama konusunda başarılı olduğumu düşünüyorum. | 1 | 2 | 3 | 4 | 5 | 6 |
| 14. Maç süresince doğru pozisyonlar alarak rakiplerime üstünlük kurabilirim. | 1 | 2 | 3 | 4 | 5 | 6 |
| **Stratejik Düşünme ve Risk Yönetimi Alt Boyutu** | | | | | | |
| 15. Maç süresince skora göre oyunun anlarını doğru oynama konusunda başarılı olduğumu düşünüyorum. | 1 | 2 | 3 | 4 | 5 | 6 |
| 16. Maç süresince ne zaman risk almam gerektiğini bilme konusunda başarılı olduğumu düşünüyorum. | 1 | 2 | 3 | 4 | 5 | 6 |
| 17. Maç süresince aldığım kararları rakibin güçlü ve zayıf yönlerine göre belirleme konusunda başarılı olduğumu düşünüyorum. | 1 | 2 | 3 | 4 | 5 | 6 |
